# fMRI spectral signatures of sleep

**DOI:** 10.1073/pnas.2016732119

**Published:** 2022-07-21

**Authors:** Chen Song, Melanie Boly, Enzo Tagliazucchi, Helmut Laufs, Giulio Tononi

**Affiliations:** ^a^Department of Psychiatry, University of Wisconsin–Madison, Madison, WI, 53719;; ^b^Cardiff University Brain Research Imaging Centre, School of Psychology, Cardiff University, Cardiff, CF24 4HQ, United Kingdom;; ^c^Department of Neurology, University of Wisconsin–Madison, Madison, WI, 53705;; ^d^Departamento de Física, Universidad de Buenos Aires and Instituto de Física de Buenos Aires, Ciudad Universitaria, Buenos Aires, 1428, Argentina;; ^e^Latin American Brain Health Institute, Universidad Adolfo Ibañez, Santiago de Chile, 3485, Chile;; ^f^Department of Neurology and Brain Imaging Center, Goethe University, Frankfurt, 60528, Germany;; ^g^Department of Neurology, University Hospital Schleswig Holstein, Kiel, 24105, Germany

**Keywords:** sleep, fMRI-EEG, BOLD oscillations, wake–sleep transitions

## Abstract

Sleep can be distinguished from wake by changes in brain electrical activity, typically assessed using electroencephalography (EEG). The hallmark of nonrapid-eye-movement (NREM) sleep is the shift from high-frequency, low-amplitude wake EEG to low-frequency, high-amplitude sleep EEG dominated by spindles and slow waves. Here we identified signatures of sleep in brain hemodynamic activity, using simultaneous functional MRI (fMRI) and EEG. We found that, at the transition from wake to sleep, fMRI blood oxygen level–dependent (BOLD) activity evolved from a mixed-frequency pattern to one dominated by two distinct oscillations: a low-frequency (<0.1 Hz) oscillation prominent in light sleep and correlated with the occurrence of spindles, and a high-frequency oscillation (>0.1 Hz) prominent in deep sleep and correlated with the occurrence of slow waves. The two oscillations were both detectable across the brain but exhibited distinct spatiotemporal patterns. During the falling-asleep process, the low-frequency oscillation first appeared in the thalamus, then the posterior cortex, and lastly the frontal cortex, while the high-frequency oscillation first appeared in the midbrain, then the frontal cortex, and lastly the posterior cortex. During the waking-up process, both oscillations disappeared first from the thalamus, then the frontal cortex, and lastly the posterior cortex. The BOLD oscillations provide local signatures of spindle and slow wave activity. They may be employed to monitor the regional occurrence of sleep or wakefulness, track which regions are the first to fall asleep or wake up at the wake–sleep transitions, and investigate local homeostatic sleep processes.

## Significance

The conventional wisdom that sleep is a global state, affecting the whole brain uniformly and simultaneously, was overturned by the discovery of local sleep, where individual neuronal populations were found to be asleep and the rest of the brain awake. However, due to the difficulty of monitoring local neuronal states in humans, our understanding of local sleep remains limited. Using simultaneous functional MRI (fMRI) and electroencephalography, we find that the oscillations of brain hemodynamic activity provide signatures of sleep at a local neuronal population level. We show that the fMRI signatures of sleep can be employed to monitor local neuronal states and investigate which brain regions are the first to fall asleep or wake up at wake–sleep transitions.

Traditionally, sleep is considered to be a global state that affects the whole brain uniformly and simultaneously. Correspondingly, brain activity during human sleep is typically measured using scalp electroencephalography (EEG). The hallmark of nonrapid-eye-movement (NREM) sleep is the shift from high-frequency, low-amplitude wake EEG to low-frequency, high-amplitude sleep EEG dominated by slow waves and spindles. Slow waves are associated with the near-synchronous transitions in large populations of neurons between depolarized up states of intense firing and hyperpolarized down states of silence (1). They are generated primarily in the cerebral cortex and affect virtually all cortical neurons, as well as neurons in several subcortical structures (2). By contrast, spindles are associated with cycles of depolarization and hyperpolarization triggered by the interactions between reticular thalamic nucleus and specific thalamic nuclei and amplified by the thalamo-cortico-thalamic circuits. Based on the prominence of slow waves and spindles, NREM sleep can be subdivided into transitional (N1), intermediate (N2), and deep (N3) sleep stages.

Recently, the view of sleep as a global state has been overturned by the intracranial findings of local sleep and local wakefulness (3). During wakefulness, individual neurons were found to display brief periods of slow wave activity, accompanied by transient behavioral impairments (4). Conversely, during deep NREM sleep, subsets of brain regions were found to display wake-like activity (5), which was associated with dreaming (6). These findings establish that sleep-like and wakefulness-like states are not mutually exclusive, but can occur simultaneously in the same brain, with some neuronal populations showing one state and the rest the other. They highlight the importance to monitor the local state of individual neuronal populations, as opposed to the global state of the brain as a whole. However, EEG lacks both the spatial resolution and the brain coverage required for monitoring local neuronal state. It is difficult to identify the brain regions that generate the scalp EEG signal, where different source configurations can give rise to the same EEG topography. Moreover, the scalp and the intracranial EEG signals are both insensitive to neuronal activities in deep brain structures, making it difficult to monitor the neuronal state in these brain regions.

Here we employed functional MRI (fMRI) to explore, with a full brain coverage and higher spatial resolution, local signatures of sleep in brain hemodynamic activity. We reasoned that the frequency content of fMRI blood oxygen level–dependent (BOLD) activity would show systematic changes from wake to sleep, reflecting the local groupings of spindles or slow waves by infra-slow fluctuations within the frequency range of brain hemodynamic activity. Our hypothesis builds upon previous reports of BOLD spectral changes from wake to sleep. Previous studies reported increases in low-frequency BOLD activity (<0.1 Hz) from wake to light sleep (7–9), as well as increases in higher-frequency BOLD activity (>0.1 Hz) from wake to propofol anesthesia (10). Although the relationships between these BOLD spectral changes and spindle or slow wave activity were not examined, it is interesting to note that propofol anesthesia can induce slow waves similar to those of NREM sleep (11), which might underlie the observed increase in high-frequency BOLD activity; moreover, the emergence of sleep spindles during child development (12) coincides with an increase in low-frequency BOLD activity (13). These studies hinted at a possible link between BOLD frequency content and spindle or slow wave activity. However, the exact link has remained unclear.

Using simultaneous fMRI and EEG, we found that, during the transition from wake to sleep, fMRI BOLD activity evolved from a mixed-frequency pattern to one dominated by two distinct oscillations: a low-frequency oscillation (<0.1 Hz) prominent in light sleep and a higher-frequency oscillation (>0.1 Hz) in deep sleep. The time courses of low-frequency and high-frequency BOLD oscillation power correlated, respectively, with the time courses of spindle and slow wave activities. Moreover, the regional distributions and the onset, offset patterns of low-frequency and high-frequency BOLD oscillation were similar to those of spindle and slow wave activity. By providing local signatures of spindle and slow wave activity, these two BOLD oscillations may be employed to monitor the local neuronal state and detect local sleep or local wakefulness.

## Results

The current study used the dataset from ref. 14. In the dataset, simultaneous fMRI and polysomnographic EEG recordings were acquired from 58 non-sleep-deprived participants, falling asleep inside a Siemens Trio 3T MRI scanner. The recordings started at around 20:00 and lasted for an hour. 36 of the 58 participants had continuous N2 and/or N3 sleep for longer than 10 min and were included in our analyses. The average time these 36 participants spent in NREM sleep was 33.27 min, and in N2 and/or N3 sleep was 22.80 min.

### Changes in BOLD Frequency Content from Wake to Sleep.

**Fig. 1.**
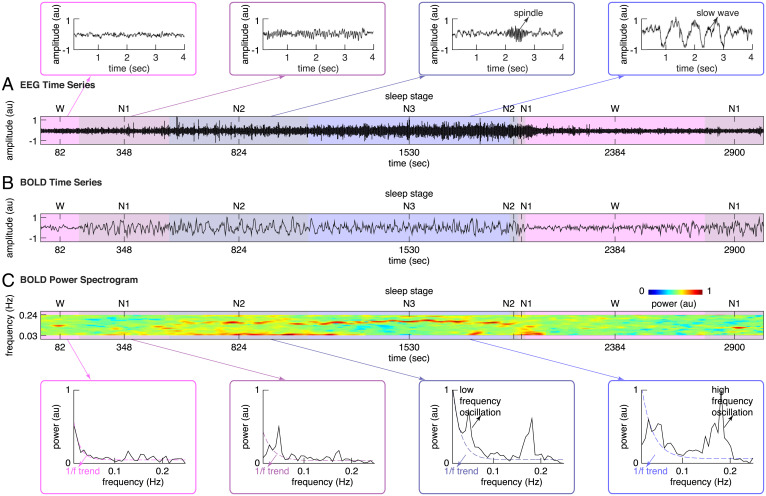
BOLD spectral changes from wake to sleep. (*A*) The EEG time series from simultaneous fMRI-EEG recordings of sleep in a representative participant were plotted, illustrating the shift from high-frequency, low-amplitude wake EEG to low-frequency, high-amplitude sleep EEG dominated by spindles and slow waves. (*B* and *C*) The BOLD time series and the BOLD power spectrogram from simultaneous fMRI-EEG recordings of sleep in the same participant were plotted, illustrating the BOLD spectral changes from wake to sleep. During wakefulness, mixed-frequency, low-amplitude BOLD activity was observed; correspondingly, the BOLD power spectrum displayed a scale-free 1/f trend. By contrast, during sleep, BOLD activity evolved into a high-amplitude background with low-frequency oscillation in N1, N2 sleep and high-frequency oscillation in N2, N3 sleep; correspondingly, the BOLD power spectrum displayed a low-frequency and a high-frequency peak on top of the scale-free 1/f trend.

Representative EEG and fMRI BOLD time series from simultaneous fMRI-EEG recordings are displayed in Fig. 1. Visual inspection of EEG time series suggested a shift from high-frequency, low-amplitude wake activity to low-frequency, high-amplitude sleep activity dominated by spindles and slow waves (Fig. 1*A*). Visual inspection of BOLD time series suggested similar, frequency-specific changes starting at the transition from wake to sleep (Fig. 1*B*). During wakefulness, mixed-frequency, low-amplitude BOLD activity was observed. By contrast, during N1 and N2 sleep, BOLD activity evolved into a high-amplitude background with prominent low-frequency oscillation. The low-frequency BOLD oscillation attenuated when reaching N3 sleep, where most brain regions started to display spontaneous BOLD oscillation of higher frequency.

To assess the progressive changes in BOLD frequency content from wake to sleep, Fast Fourier Transform (FFT) analysis was applied to pre-processed BOLD time series in sliding Hamming windows of 104 s (50 volumes) and step sizes of 2.08 s (1 volume). The BOLD power spectrogram derived from sliding-window FFT analysis confirmed our visual detection of a low-frequency BOLD oscillation emerging during N1 and N2 sleep, which progressively attenuated when transitioning to N3 sleep (Fig. 1*C*). It also revealed that the higher-frequency BOLD oscillation visually detected in N3 sleep was already present, although at a lower power, during N1 and N2 sleep (Fig. 1*C*).

Based on the BOLD power spectrogram, we calculated the BOLD power spectrum for wake, N1, N2, and N3 sleep. Consistent with the literatures (15), the BOLD power spectrum during wakefulness displayed a scale-free 1/f trend (Fig. 1*C*), confirming the visual inspection of a mixed-frequency pattern. By contrast, the BOLD power spectrum during NREM sleep displayed a low-frequency peak (<0.1 Hz, mostly around 0.04∼0.07 Hz) and a higher-frequency peak (>0.1 Hz, mostly around 0.15∼0.18 Hz) on top of the scale-free 1/f trend (Fig. 1*C*).

### Regional Distributions of BOLD Oscillations during Sleep.

**Fig. 2.**
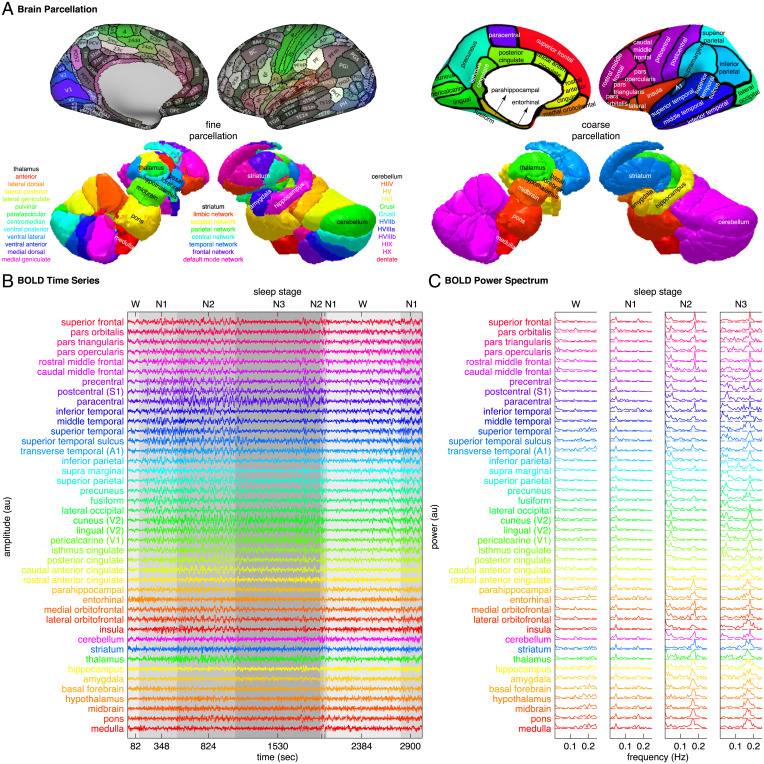
BOLD spectral analysis. (*A*) To investigate the regional distributions of BOLD oscillations, we parcellated the cortex into 32 coarse regions or 180 fine regions, and the subcortex into 10 coarse regions (cerebellum, striatum, thalamus, medulla, pons, midbrain, hypothalamus, basal forebrain, amygdala, and hippocampus) or 37 fine regions (11 cerebellum lobules, 7 striatum divisions, 12 thalamic subregions, medulla, pons, midbrain, hypothalamus, basal forebrain, amygdala, and hippocampus). (*B* and *C*) We derived the BOLD power spectrogram of each brain region by applying sliding-window FFT analysis to the BOLD time series of individual voxels and computing the average power spectrogram across all voxels within the region. Plotted here are the regional average BOLD time series and the regional average BOLD power spectrum from simultaneous fMRI-EEG recordings of sleep in a representative participant.

The changes in BOLD frequency content from wake to sleep were observed at a local, voxel level across the brain, but with regional differences in oscillation power and oscillation frequency (Fig. 2). To investigate the regional distributions of BOLD oscillations, we parcellated the cortex into 32 coarse regions or 180 fine regions, and the subcortex into 10 coarse regions or 37 fine regions (Fig. 2*A*). We derived the BOLD power spectrogram of each brain region by applying sliding-window FFT analysis to the BOLD time series of individual voxels and computing the average power spectrogram across all voxels within the region; based on the BOLD power spectrogram, we identified the peak frequencies of BOLD oscillations and traced the power of BOLD oscillations[Sec s11].

**Fig. 3.**
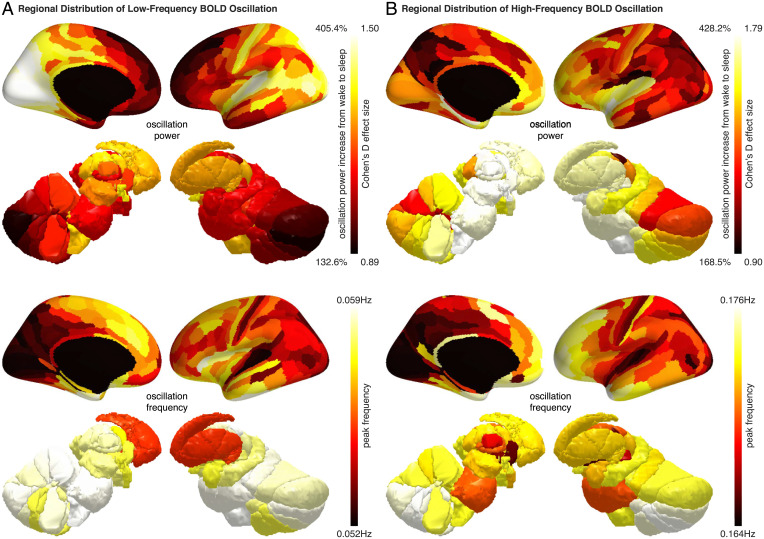
Regional distributions of BOLD oscillations during sleep. Regional increase in BOLD oscillation power from wake to sleep, averaged across two hemispheres and all participants, was projected onto three-dimensional brain models (*Upper*). The power of low-frequency BOLD oscillation (*A*) and the power of high-frequency BOLD oscillation (*B*) both showed a 200 to 300% increase from wake to sleep. However, the low-frequency BOLD oscillation was strong in posterior and sensory regions but weak in frontal and entorhinal regions (*A*), whereas the high-frequency BOLD oscillation was strong in frontal and entorhinal regions but weak in posterior and sensory regions (*B*). Regional value of BOLD oscillation frequency, averaged across two hemispheres and all participants, was also projected onto three-dimensional brain models (*Lower*). The oscillation frequency was higher in frontal and subcortical regions and lower in posterior and sensory regions for both the low-frequency BOLD oscillation (*A*) and the high-frequency BOLD oscillation (*B*).

From wake to sleep, the low-frequency and the high-frequency BOLD oscillations both showed a 200 to 300% increase in power. The magnitude of the increase was substantial, considering that the increase in BOLD amplitude evoked by stimuli or tasks during wakefulness is usually less than 10% (16). Although the increase in BOLD oscillation power from wake to sleep was observed across the brain, the exact magnitude of the increase differed between brain regions. Within the cortex, the power of low-frequency BOLD oscillation increased by up to 400% in posterior and sensory regions, including the visual, auditory, somatosensory, precuneus, and posterior cingulate areas, but by only 150% in frontal and entorhinal regions (Fig. 3*A*); the power of high-frequency BOLD oscillation, on the other hand, increased by up to 425% in frontal and entorhinal regions, but by only 200% in posterior and sensory regions (Fig. 3*B*). Within the subcortex, the low-frequency and the high-frequency BOLD oscillations had more similar regional distributions. Both showed the highest increase of oscillation power in the hypothalamus, basal forebrain, and the intralaminar, anterior, medial dorsal, lateral dorsal thalamus (Fig. 3).

Similar to the power of BOLD oscillations, the peak frequencies of BOLD oscillations exhibited regional differences. For both the low-frequency and the high-frequency BOLD oscillations, the peak frequencies were slightly higher in frontal and subcortical regions, and lower in posterior and sensory regions (Fig. 3). The regional differences in oscillation power and oscillation frequency suggested that the BOLD oscillations might provide local signatures of sleep.

### BOLD Oscillations Provide Signatures of Spindle and Slow Wave Activity.

Consistent with the hypothesis that BOLD oscillations provide signatures of sleep, the time courses of the two BOLD oscillations mirrored these of sleep spindles and slow waves. Specifically, the low-frequency BOLD oscillation was prominent in N2 sleep and the high-frequency BOLD oscillation in N3 sleep, mirroring the prominence of spindles in N2 sleep and slow waves in N3 sleep. Moreover, the peak frequencies of the two BOLD oscillations, 0.04∼0.07 Hz and 0.15∼0.18 Hz, were similar to the reported periodicity of spindles (around two spindles per minute, falling in the range of 0.04∼0.07 Hz) ( 17) and slow waves (around seven slow waves per minute, falling in the range of 0.15∼0.18 Hz) (18).

**Fig. 4.**
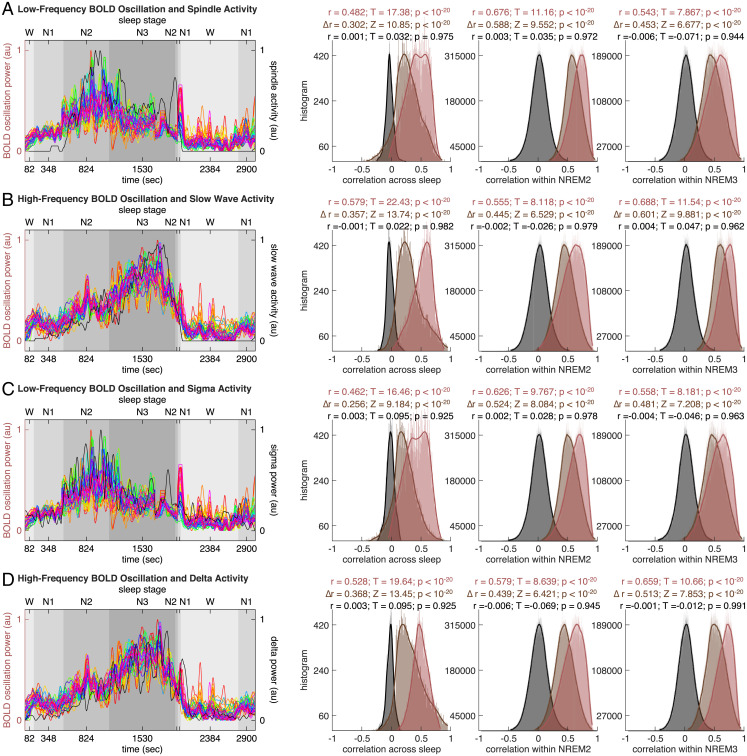
BOLD oscillations and spindle or slow wave activity. The time course of low-frequency BOLD oscillation power (*A* and *C*, colored lines, one color per region, see Fig. 2 for color codes) or high-frequency BOLD oscillation power (*B* and *D*, colored lines, one color per region, see Fig. 2 for color codes) or BOLD amplitude was correlated against the time course of spindle activity (*A*, black line), slow wave activity (*B,* black line), sigma power (*C*, black line), or delta power (*D*, black line), both across sleep stages (examining global, across-stage correlation) and within sleep stages (examining local, within-stage correlation). The correlation was calculated on a region-by-region, participant-by-participant basis. The distribution of correlation coefficient across all brain regions and all participants was plotted, to evaluate the statistical significance of the correlation. The analysis revealed a positive correlation between low-frequency BOLD oscillation and spindle activity (*A*, red-colored histograms) or sigma activity (*C*, red-colored histograms), as well as a positive correlation between high-frequency BOLD oscillation and slow wave activity (*B*, red-colored histograms) or delta activity (*D*, red-colored histograms). It also revealed a lack of correlation between BOLD amplitude and spindle activity (*A*, gray-colored histograms), slow wave activity (*B*, gray-colored histograms), sigma activity (*C*, gray-colored histograms), or delta activity (*D*, gray-colored histograms). Moreover, it showed that the low-frequency BOLD oscillation correlated more strongly with spindle activity than with slow wave activity (*A*, brown-colored histograms), and with sigma activity than with delta activity (*C*, brown-colored histograms), whereas the high-frequency BOLD oscillation correlated more strongly with slow wave activity than with spindle activity (*B*, brown-colored histograms), and with delta activity than with sigma activity (*D*, brown-colored histograms).

To examine the relationships between BOLD oscillations and spindle or slow wave activity, we derived the time course of spindle or slow wave activity from EEG data and correlated that against the time course of BOLD oscillation power[Sec s11]. Specifically, we detected individual occurrences of spindles or slow waves using methods described in refs. 19, 20 and calculated spindle or slow wave activity as the integral of their occurrence and their duration in consecutive windows of 2.08 s (matching fMRI temporal resolution). Since individual data points in the time course of BOLD oscillation power were derived from sliding-window FFT analyses as the weighted average of BOLD oscillation power in 104-s Hamming windows, we derived individual data points in the time course of spindle or slow wave activity in a corresponding way as the weighted average of spindle or slow wave activity in 104-s Hamming windows. We then correlated the time course of spindle or slow wave activity against the time course of BOLD oscillation power, both across sleep stages (examining global, across-stage correlation) and within sleep stages (examining local, within-stage correlation). The correlation was calculated on a region-by-region, participant-by-participant basis. The distribution of correlation coefficient across all brain regions and all participants was plotted to evaluate the statistical significance of the correlation (Fig. 4).

The analysis revealed a positive correlation between the time course of low-frequency BOLD oscillation power and the time course of spindle activity, both across sleep stages and within sleep stages (Fig. 4*A*). It also revealed a positive correlation between the time course of high-frequency BOLD oscillation power and the time course of slow wave activity (Fig. 4*B*). Moreover, the low-frequency BOLD oscillation power correlated more strongly with spindle than with slow wave activity (Fig. 4*A*), whereas the high-frequency BOLD oscillation power correlated more strongly with slow wave than with spindle activity (Fig. 4*B*). Since spindle and slow wave activities can be approximated, respectively, by EEG sigma (11∼16 Hz) and delta (0.5∼4 Hz) band power, we further examined the relationships between BOLD oscillation power and sigma or delta power. To this end, FFT analysis was applied to EEG time series in sliding Hamming windows of 104 s and step sizes of 2.08 s (matching the window size and the step size of FFT analysis to BOLD time series), from which we derived the EEG power spectrogram, traced the time course of sigma or delta power, and correlated that against the time course of BOLD oscillation power. We observed a positive correlation between the time course of low-frequency BOLD oscillation power and the time course of sigma power (Fig. 4*C*), as well as a positive correlation between the time course of high-frequency BOLD oscillation power and the time course of delta power (Fig. 4*D*). Also, the low-frequency BOLD oscillation power correlated more strongly with sigma than with delta power (Fig. 4*C*), whereas the high-frequency BOLD oscillation power correlated more strongly with delta than with sigma power (Fig. 4*D*).

In contrast to the frequency content, the amplitude of BOLD activity did not show significant correlation with spindle (Fig. 4*A*) or slow wave activity (Fig. 4*B*), and neither did it show significant correlation with sigma (Fig. 4*C*) or delta power (Fig. 4*D*). Together these results suggested that the BOLD frequency content, as opposed to the BOLD amplitude, provided signatures of spindles and slow waves.

### Onset and Offset of BOLD Oscillations at Wake–Sleep Transitions.

Notably, while the changes in BOLD frequency content at the wake–sleep transitions were observed across the brain, the onset or offset of these BOLD spectral changes differed between brain regions, with some regions showing an earlier onset of BOLD oscillations than other regions during the falling asleep process, or an earlier offset of BOLD oscillations than other regions during the waking up process. To estimate the time lags between brain regions in the onset of BOLD oscillations (during the falling asleep process) or the offset of BOLD oscillations (during the waking up process), time-lagged cross-correlation analysis was applied to the time courses of BOLD oscillation power [Sec s11](21).

**Fig. 5.**
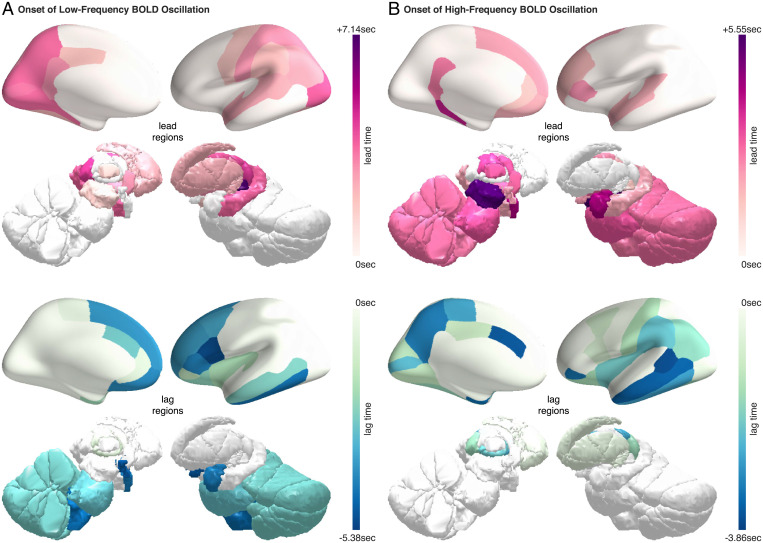
Onset of BOLD oscillations during the falling asleep process. The temporal lag between different brain regions in the onset of BOLD oscillations at the transition from wake to sleep was projected onto three-dimensional brain models. The regions that led other regions in the onset of BOLD oscillations are shown in red-purple colormap (*Upper*), where the lead time quantifies the degree to which they led. The regions that lagged behind other regions in the onset of BOLD oscillations are shown in blue-green colormap (*Lower*), where the lag time quantifies the degree to which they lagged. For better illustrations, the lead time and the lag time are marked with plus and the minus signs, respectively. The low-frequency BOLD oscillation and the high-frequency BOLD oscillation had markedly distinct onset patterns. (*A*) The low-frequency BOLD oscillation first appeared in the sensory thalamus and then in sensory and posterior cortices; the frontal cortices, by comparison, were among the last regions to see the onset of low-frequency BOLD oscillation. (*B*) The high-frequency BOLD oscillation, on the other hand, first appeared in the midbrain and then in frontal cortices. The sensory and posterior cortices, by comparison, were among the last regions to see the onset of high-frequency BOLD oscillation.

The analysis revealed that, during the falling asleep process, the low-frequency BOLD oscillation first appeared in the sensory thalamus (lateral geniculate and medial geniculate thalamus) and then in other thalamic subregions (Fig. 5*A*). Soon after, the oscillation appeared in the cortex and specifically in the posterior and sensory regions. The frontal cortex, by comparison, was among the last regions to have the onset of low-frequency BOLD oscillation. In contrast to the onset pattern of low-frequency BOLD oscillation, the high-frequency BOLD oscillation first appeared in the midbrain, followed by other subcortical regions, including the thalamus, amygdala, pons, medulla, basal forebrain, and hypothalamus (Fig. 5*B*). The oscillation then appeared in the cortex and specifically in the frontal regions. The posterior and sensory cortices, by comparison, were among the last regions to have the onset of high-frequency BOLD oscillation.

**Fig. 6.**
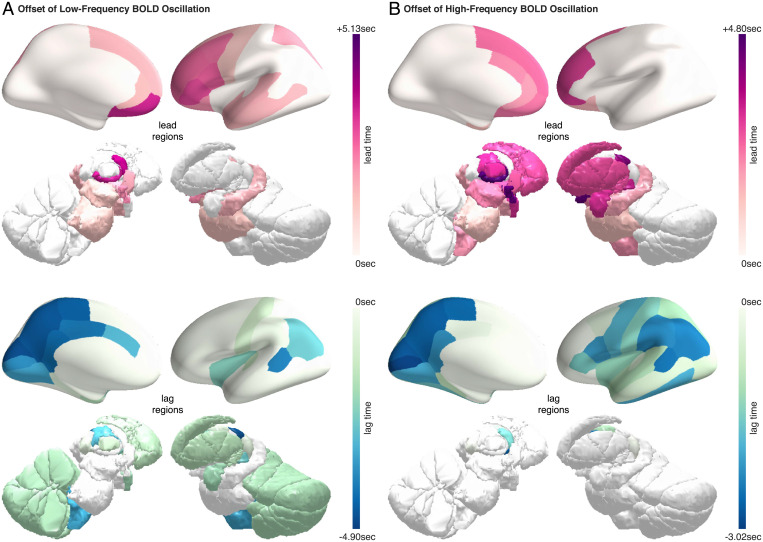
Offset of BOLD oscillations during the waking up process. The temporal lag between different brain regions in the offset of BOLD oscillations at the transition from sleep to wake was projected onto three-dimensional brain models. The regions that led other regions in the offset of BOLD oscillations are shown in red-purple colormap (*Upper*), where the lead time quantifies the degree to which they led. The regions that lagged behind other regions in the offset of BOLD oscillations are shown in blue-green colormap (*Lower*), where the lag time quantifies the degree to which they lagged. For better illustrations, the lead time and the lag time are marked with plus and the minus signs, respectively. The low-frequency BOLD oscillation (*A*) and the high-frequency BOLD oscillation (*B*) had similar offset patterns. They both disappeared first from the intralaminar thalamus and then from other thalamic subregions; soon after, they disappeared in the cortex, starting with frontal regions and ending with posterior and sensory regions.

Whereas the low-frequency and the high-frequency BOLD oscillations had different onset patterns, they had similar offset patterns. During the waking up process, the low-frequency BOLD oscillation disappeared first from the intralaminar thalamus and then from other thalamic subregions (Fig. 6*A*). Soon after, the oscillation disappeared in the cortex, starting with frontal regions and ending with posterior and sensory regions. Similarly, the high-frequency BOLD oscillation disappeared first from the intralaminar thalamus, followed by other thalamic subregions (Fig. 6*B*). The oscillation then disappeared in the cortex, also starting with frontal regions and ending with posterior and sensory regions. The gradual onset of BOLD oscillations at the transition from wake to sleep, and the gradual offset of BOLD oscillations at the transition from sleep to wake, indicated a lack of synchronization between brain regions in their local states. Possibly, the order of onset (during the falling asleep process) and the order of offset (during the waking up process) reflected which brain regions were the first to fall asleep and the first to wake up, respectively.

### Impacts of Physiological Activities.

Since fMRI BOLD activity is the product of a complex interplay between neuronal and vascular events, the observation of BOLD oscillations during sleep could be driven by non-neuronal physiological signals (22). To control for non-neuronal physiological impacts, an extensive set of physiological regressors was derived from the raw respiratory and cardiac data collected during fMRI-EEG recordings, including respiratory phase, respiratory rate, respiratory volume, respiratory depth, cardiac phase, and cardiac rate (23–25). The set of physiological regressors was regressed out from BOLD time series during fMRI pre-processing [Sec s11](26), before FFT analysis was applied to assess the BOLD frequency content. As such, the BOLD oscillations reported in our study are unlikely to result merely from non-neuronal physiological signals.

**Fig. 7.**
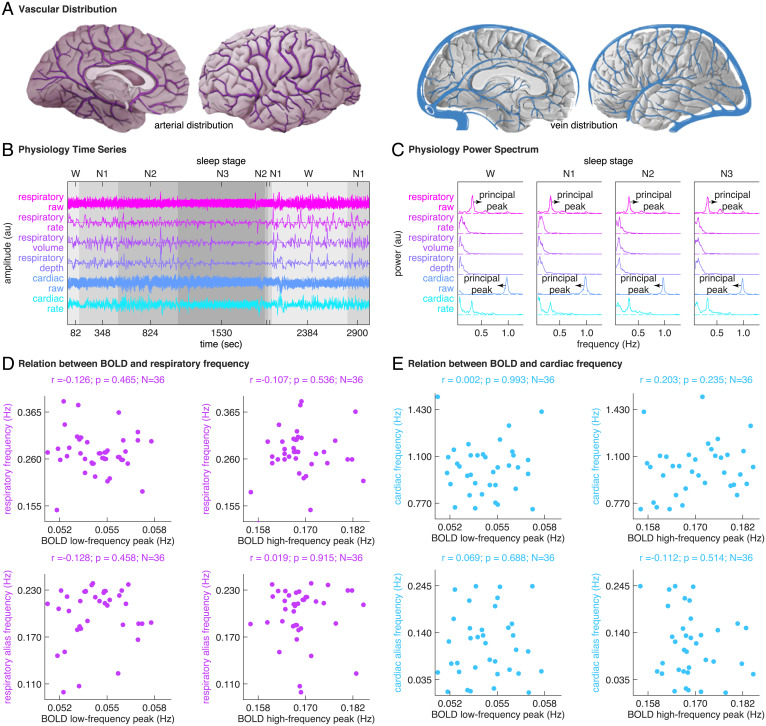
Physiology spectral analysis. (*A*–*C*) To examine whether the pre-processed fMRI data were indeed clean of non-neuronal physiological signals, we inspected the frequency content of respiratory or cardiac activity and compared that against the BOLD frequency content. We applied sliding-window FFT analysis to the time series of raw respiratory data, respiratory rate, respiratory volume, respiratory depth, raw cardiac data, and cardiac rate. The power spectrum of raw respiratory data had a principal peak at around 0.25 Hz and the power spectrum of raw cardiac data had a principal peak at around 1 Hz, reflecting, respectively, the oscillations in respiration and cardiac pulse activities. The power spectrums of respiratory rate, respiratory volume, respiratory depth, and cardiac rate can be divided into the low-frequency (0.04∼0.15 Hz) and high-frequency (0.15∼0.4 Hz) bands, reflecting, respectively, the slower and faster oscillations in these physiological signals. (*D* and *E*) Based on the power spectrums of raw respiratory data and raw cardiac data, we extracted the respiration and cardiac pulse frequencies, calculated their alias frequencies, and compared these physiological frequencies against the peak frequencies of BOLD oscillations, on an interindividual basis across all participants. We did not observe a significant correlation between BOLD oscillation frequencies and respiratory or cardiac frequencies.

Nonetheless, it was important to examine whether the pre-processed fMRI data were indeed clean of non-neuronal physiological signals. To this end, FFT analysis was applied to the time series of raw respiratory data, respiratory rate, respiratory volume, respiratory depth, raw cardiac data, and cardiac rate (Fig. 7*B*) in sliding Hamming windows of 104 s and step sizes of 2.08 s (matching the window size and the step size of FFT analysis to BOLD time series), in order to inspect the frequency content of respiratory or cardiac activity and compare that against the BOLD frequency content. The power spectrum of raw respiratory data had a principal peak at around 0.25 Hz and the power spectrum of raw cardiac data had a principal peak at around 1 Hz, reflecting, respectively, the oscillations in respiration and cardiac pulse activities (Fig. 7*C*) (27, 28). The power spectrums of respiratory rate, respiratory volume, respiratory depth, and cardiac rate can be divided into the low-frequency (0.04∼0.15 Hz) and the high-frequency (0.15∼0.4 Hz) bands, reflecting, respectively, the slower and faster oscillations in these physiological signals (Fig. 7*C*) (29, 30).

**Fig. 8.**
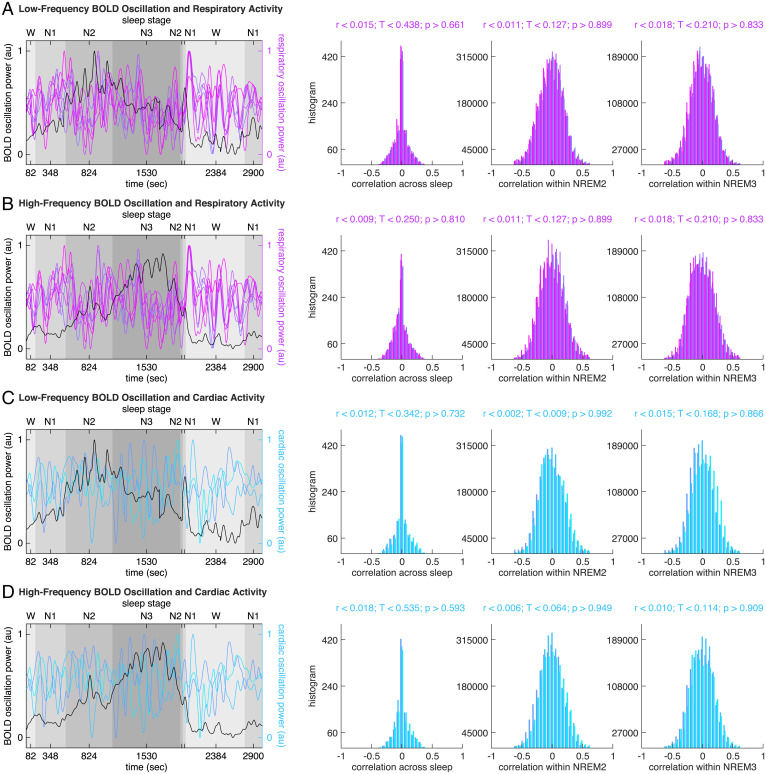
BOLD oscillations and respiratory or cardiac activity. The time course of low-frequency BOLD oscillation power (*A* and *C*, black lines, illustrating whole brain average) or high-frequency BOLD oscillation power (*B* and *D*, black lines, illustrating whole brain average) was correlated against the time course of respiratory oscillation power (*A* and *B*, colored lines, see Fig. 7 for color codes), including the band-limited power of respiration activity (respiration frequency ± 0.1 Hz), the low-frequency (0.04∼0.15 Hz) and high-frequency (0.15∼0.4 Hz) power of respiratory rate, respiratory volume, respiratory depth, or the time course of cardiac oscillation power (*C* and *D*, colored lines, see Fig. 7 for color codes), including the band-limited power of cardiac pulse activity (cardiac pulse frequency ± 0.4 Hz), the low-frequency (0.04∼0.15 Hz) and high-frequency (0.15∼0.4 Hz) power of cardiac rate , both across sleep stages (examining global, across-stage correlation) and within sleep stages (examining local, within-stage correlation). The correlation was calculated on a region-by-region, participant-by-participant basis. The distribution of correlation coefficient across all brain regions and all participants was plotted to evaluate the statistical significance of the correlation. The analysis revealed a lack of correlation between BOLD oscillation power and respiratory or cardiac oscillation power.

Based on the power spectrums of raw respiratory data and raw cardiac data, we extracted the respiration and cardiac pulse frequencies, calculated their alias frequencies, and compared these physiological frequencies against the peak frequencies of BOLD oscillations (Fig. 7). Based on the power spectrums of raw respiratory data, raw cardiac data, respiratory rate, respiratory volume, respiratory depth, and cardiac rate, we extracted the band-limited power of respiration activity (respiration frequency ± 0.1 Hz), the band-limited power of cardiac pulse activity (cardiac pulse frequency ± 0.4 Hz), the low-frequency (0.04∼0.15 Hz) and the high-frequency (0.15∼0.4 Hz) power of respiratory rate, respiratory volume, respiratory depth, and cardiac rate. We then correlated the time courses of these physiological powers against the time courses of BOLD oscillation power, both across sleep stages (examining global, across-stage correlation) and within sleep stages (examining local, within-stage correlation). The correlation was calculated on a region-by-region, participant-by-participant basis. The distribution of correlation coefficient across all brain regions and all participants was plotted to evaluate the statistical significance of the correlation (Fig. 8).

From the analyses we did not observe significant correlation between BOLD oscillation frequencies and respiratory (Fig. 7*D*) or cardiac (Fig. 7*E*) frequencies, or between the time course of BOLD oscillation power and the time course of respiratory or cardiac oscillation power (Fig. 8). In addition to comparing the temporal characteristics, we also compared the spatial characteristics of BOLD oscillations and physiological activities. We reasoned that if BOLD oscillations were driven by non-neuronal physiological signals, the spatial distributions of BOLD oscillations would resemble vascular distributions in the brain ( 31). For example, the BOLD oscillations would be strong in the occipital pole (confluence of sinuses), superior frontal cortex (superior sagittal sinus), but weak in the orbitofrontal cortex. We did not observe similarity between the spatial distributions of BOLD oscillations (Fig. 3) and the vascular distributions in the brain (Fig. 7*A*). These control analyses suggested that the BOLD oscillations reported in our study were not driven primarily by non-neuronal physiological signals.

## Discussion

Taken together, we found that during the transition from wake to sleep, fMRI BOLD activity evolved from a mixed-frequency pattern to one dominated by two distinct oscillations: a low-frequency (<0.1 Hz) oscillation prominent in light sleep and a higher-frequency oscillation (>0.1 Hz) in deep sleep. The two oscillations were detectable at a local, voxel level across the brain, with their spatiotemporal distributions mirroring those of spindle and slow wave activity. The oscillations likely reflect the local groupings of spindles or slow waves by infra-slow fluctuations within the frequency range of brain hemodynamic activity. They provide local signatures of spindle and slow wave activity, which may be employed to monitor local neuronal state and detect local sleep or local wakefulness.

### BOLD Oscillations Mirror Spindle and Slow Wave Activities.

In line with our findings, previous studies have reported BOLD spectral changes from wake to sleep (7–9). However, the properties of these changes and their relations to spindle or slow wave activity have remained unclear. Here our analysis revealed positive correlations between the time course of low-frequency BOLD oscillation power and the time course of spindle activity, and between the time course of high-frequency BOLD oscillation power and the time course of slow wave activity. Moreover, the regional distributions as well as the onset and the offset patterns of the low-frequency and high-frequency BOLD oscillations mirrored these of spindle and slow wave activity.

During sleep, the low-frequency and the high-frequency BOLD oscillations were detected in all cortical and subcortical regions examined, consistent with the widespread detection of spindles and slow waves in intracranial recordings (32–36). The regional distribution of low-frequency BOLD oscillation mirrored that of spindle activity, with large-amplitude spindles reported in thalamus, sensory, and orbitofrontal cortices, but reduced occurrence of spindles in parahippocampal cortex (34–37). The regional distribution of high-frequency BOLD oscillation, on the other hand, mirrored that of slow wave activity, and some of the regions showing the strongest high-frequency BOLD oscillation, such as medial prefrontal cortex, parahippocampal cortex, and brainstem, were also highlighted in previous fMRI studies of slow waves (38, 39). The subgenual area in the orbitofrontal cortex is notable for showing strong BOLD oscillations in both the low-frequency and the high-frequency ranges. Intriguingly, the orbitofrontal cortex was found to have among the highest amplitudes of spindles (37); it is also involved in the origin and propagation of slow waves (40, 41).

During the falling asleep process, the low-frequency BOLD oscillation first appeared in the thalamus. This result is reminiscent of the intracranial finding that the inactivation of the thalamus precedes that of the cortex at the transition from wake to sleep (42). The low-frequency BOLD oscillation then appeared in the cortex and specifically in the posterior regions. The earlier onset of low-frequency BOLD oscillation in posterior regions compared to frontal regions mirrors the onset pattern of spindles, where parietal spindles were found to appear before frontal spindles (43). The high-frequency BOLD oscillation, on the other hand, first appeared in the midbrain. Intriguingly, a unit recording study of the midbrain reticular formation neurons in sleeping cats has found rhythmic fluctuations in neuronal firing rates, phase locked to the increases in amplitudes of cortical slow waves during sleep (44); the rhythmic fluctuations had a modal period of ∼11 s, corresponding exactly to the high-frequency BOLD oscillation (0.15∼0.18 Hz). The high-frequency BOLD oscillation then appeared in the cortex and specifically in the frontal regions. This pattern mirrors the onset of slow waves, which were found to appear in frontal regions before other cortical regions (45).

During the waking up process, the intralaminar thalamus was the first brain region to show the offset of both the low-frequency and the high-frequency BOLD oscillations. In animals, stimulation of intralaminar thalamic nuclei can reliably produce awakenings from sleep (46, 47). Human data also indicate a central role of the intralaminar thalamus in the regulation of arousal, as part of an anterior forebrain circuit involving both subcortical and frontal regions (48, 49). In addition to the intralaminar thalamus, other brain regions involved in arousal, such as the hypothalamus and basal forebrain (50), were also among the first regions to show an offset of both the low-frequency and the high-frequency BOLD oscillations at the transition from sleep to wake.

### Potential Mechanisms underlying BOLD Oscillations.

The similarities between the spatiotemporal distributions of BOLD oscillations and the spatiotemporal characteristics of spindle and slow wave activity suggest that the BOLD oscillations provide local signatures of sleep. However, it should be emphasized that the detection of BOLD oscillations during sleep cannot be expected to have a one-to-one correspondence with the occurrence of individual spindles or slow waves. What the data show is simply that the average BOLD oscillation power in 104-s Hamming windows correlates with the average spindle or slow wave activity. The correlations may be established through the local groupings of spindles or slow waves by infraslow fluctuations within the frequency range of brain hemodynamic activity.

During sleep, the membrane potential of neurons undergoes periodic fluctuations, reflected in the EEG as spindles and slow waves. These fluctuations in membrane potential involve the movements of ions across the cell membrane, and the restoration of ionic concentration, in turn, requires the supply of metabolic energy in the form of adenosine triphosphate (ATP). As the brain does not store ATP, it must synthesize ATP on site through the oxidation of glucose, supplied by an increase in blood flow. During this process, deoxyhemoglobin is flushed out from blood vessels. Being paramagnetic, its movement in the magnetic field alters the spins of nearby water molecules, which can lead to a local change in BOLD activity. Thus, the spontaneous oscillations in BOLD activity are likely to result from the fluctuations in the membrane potential of neurons (51), the same cellular events picked up by the EEG as spindles and slow waves. The mechanisms responsible for the much slower modulation of neuronal excitability in the range of the high- and low-frequency BOLD oscillations reported here remain to be determined. However, the demonstration of such BOLD oscillations in subcortical areas suggests the possibility that both neuronal excitability and other physiological variables, including those involved in vegetative functions, may be influenced by rhythmic processes occurring in brainstem regions.

In our study, both the low-frequency and the high-frequency BOLD oscillations were detected in subcortical regions, such as the brainstem and cerebellum, where spindles and slow waves have not been previously recorded. It is possible that the presence of BOLD oscillations in these regions is a signature of the occurrence of genuine spindles or slow waves that had not yet been documented due to limitations of intracranial or scalp recording. A second possibility is that the BOLD oscillations detected in these regions may reflect fluctuations in neuronal excitability that do not necessarily result in spindles or slow waves. For example, diffuse subcortical projections from midbrain reticular formation may not only affect the likelihood of neurons in the cortex entering depolarized up states or hyperpolarized down states, leading to changes in slow wave or spindle activity there, but also may affect the fluctuations in neuronal excitability in brainstem or cerebellar regions where neurons may lack the mechanisms for producing spindles and slow waves. As a third possibility, in brain regions that lack the mechanisms for spindle or slow wave generation, the BOLD oscillations may reflect the synaptic input synchronized with the occurrence of spindles or slow waves elsewhere in the brain. Indeed, cortical slow waves, which involve the synchronous down states across large populations of neurons, are bound to impose a strong modulation, directly or indirectly, on many subcortical regions, including the brainstem and cerebellum (52).

### Future Perspectives.

The current study was based on an archival dataset (14). Whereas this dataset had a large number of participants and a natural induction of sleep, it was limited in several aspects. On the sleep side, the absence of adaptation nights and the short duration of sleep inside the MRI scanner did not allow participants to enter REM sleep. On the fMRI side, the relatively low temporal resolution limited the coverage of BOLD frequencies. Using fast fMRI (53), future studies may expand the coverage of BOLD frequencies. Moreover, by introducing adaptation nights and achieving longer recording of sleep inside the MRI scanner (54), future studies may investigate how the BOLD oscillations change over the course of sleep and whether the oscillations exhibit trait-like inter-individual variability. Spindle activity is known to exhibit trait-like inter-individual variability that correlates with inter-individual differences in cognition (55). Slow wave activity, on the other hand, is known to be homeostatically regulated, decreasing over the course of sleep (20, 56) and correlating with the cognitive improvements after sleep (57). These findings, based on EEG measures, could not readily address the roles different brain regions play in the links between sleep and cognition. By comparison, the fMRI signatures of sleep may allow a better characterization of the sleeping brain processes and a finer understanding of the links between sleep and cognition.

More importantly, the fMRI signatures of sleep provide a potential tool for monitoring local neuronal state and studying the interactions between local neuronal state and global brain state. The gradual onset of BOLD oscillations (during the falling asleep process) or offset of BOLD oscillations (during the waking up process) point to a lack of synchronization between brain regions in their local states. During the falling asleep process, the onset of high-frequency BOLD oscillation started subcortically in midbrain and cortically in frontal regions, mirroring the onset pattern of slow waves, while the onset of low-frequency BOLD oscillation started subcortically in thalamus and cortically in parietal regions, mirroring the onset pattern of spindles. Since slow waves have an earlier onset than spindles at the transition from wake to sleep (45), our finding suggests that the regions showing the earliest onset of slow waves or high-frequency BOLD oscillation, such as frontal regions, may be among the first regions to fall asleep. During the waking up process, the offset of high-frequency and low-frequency BOLD oscillations started subcortically in the thalamus and cortically in frontal regions, suggesting that frontal regions may be among the first regions to wake up. This observation seems at variance with previous evidence that frontal regions are among the last regions to wake up (58, 59). However, future research is needed to investigate whether the onset or offset pattern reported in our study reflects first-night effect induced by MRI sleep environment or reflects more general sleeping pattern. Future research is also needed to explore whether the onset of local sleep or wakefulness follows a fixed sequence or is homeostatically regulated, starting from the regions with the highest sleep pressure (the onset of local sleep) or the lowest sleep pressure (the onset of local wakefulness).

Furthermore, the fMRI signatures of sleep provide a potential means to study the functions of local sleep or local wakefulness. Due to the difficulty of detecting local sleep or local wakefulness in human participants, little is known about their functional roles. It is not known whether local sleep is detrimental, by causing transient behavioral deficits, or is instead beneficial and plays a restorative role. It is also not known whether local wakefulness is detrimental, for example by preventing neuronal populations from functional restoration, or is instead beneficial and plays an adaptative role. Future studies may employ fMRI to detect the occurrence of local sleep or local wakefulness in human participants and study the functional roles they play.

## Methods

See SI for the details of methods, including Data Acquisition, MRI Brain Parcellation, FMRI Spectral Analysis, FMRI Time-Lagged Cross-Correlation Analysis, Physiological Noise Correction, Physiological Spectral Analysis, EEG Analysis, and Multimodal Correlation Analysis.

## Data Availability

This work was based on a previously published dataset (14). The data analysis scripts and toolboxes have been deposited in Zenodo (60).
